# “It’s Worse to Breathe It Than to Smoke It”: Secondhand Smoke Beliefs in a Group of Mexican and Central American Immigrants in the United States

**DOI:** 10.3390/ijerph17228630

**Published:** 2020-11-20

**Authors:** Thomas A. Arcury, Grisel Trejo, DaKysha Moore, Timothy D. Howard, Sara A. Quandt, Edward H. Ip, Joanne C. Sandberg

**Affiliations:** 1 Department of Family and Community Medicine, Wake Forest School of Medicine, Winston-Salem, NC 27157, USA; gtrejo@wakehealth.edu (G.T.); jsandber@wakehealth.edu (J.C.S.); 2 Department of Visual, Performing, & Communication Arts, Johnson C. Smith University, Charlotte, NC 28216, USA; damoore@jcsu.edu; 3 Department of Biochemistry, Wake Forest School of Medicine, Winston-Salem, NC 27157, USA; tdhoward@wakehealth.edu; 4 Department of Epidemiology and Prevention, Wake Forest School of Medicine, Winston-Salem, NC 27157, USA; squandt@wakehealth.edu; 5 Department of Biostatistics and Data Science, Wake Forest School of Medicine, Winston-Salem, NC 27157, USA; eip@wakehealth.edu

**Keywords:** tobacco control and prevention, environmental tobacco smoke, minority health, health beliefs, ethnomedicine

## Abstract

This analysis describes beliefs about secondhand smoke and its health effects held by Mexican and Central American immigrants in North Carolina. Data from 60 semistructured, in-depth interviews were subjected to saliency analysis. Participant discussions of secondhand smoke centered on four domains: (1) familiarity and definition of secondhand smoke, (2) potency of secondhand smoke, (3) general health effects of secondhand smoke, and (4) child health effects of secondhand smoke. Secondhand smoke was generally believed to be more harmful than primary smoke. Mechanisms for the potency and health effects of secondhand smoke involved the smell of secondhand smoke, secondhand smoke being an infection and affecting the immune system, and personal strength being protective of secondhand smoke. Understanding these health beliefs informs a framework for further health education and intervention to reduce smoking and secondhand smoke exposure in this vulnerable population.

## 1. Introduction

Secondhand smoke (environmental tobacco smoke) is a medical and public health concern because exposure increases the risk for respiratory diseases, coronary heart diseases, cancer, and stroke [1,2,3]. Secondhand smoke exposure is of particular concern for those living in low-income and minority communities [4,5,6]. Higher rates of smoking, greater residential density, and less control on non-smoking enforcement increase the risk of secondhand smoke exposure in these communities [7,8,9]. Black Americans report greater secondhand smoke exposure than do Whites, Asians, or Latinos [6,10,11]. However, nearly one-quarter (23.9%) of Mexican American nonsmokers were exposed to secondhand smoke [2].

An accurate understanding of community knowledge and beliefs about secondhand smoke is important for successful health and public health communication and practice [12]. This understanding is especially important when the community includes immigrants who do not speak the dominant language and who have different life experiences. This is the case for many Latinx immigrants in the United States (US).

Details of Latinx knowledge and beliefs surrounding secondhand smoke are limited. Several important analyses have examined some aspects of Latinx perceptions surrounding secondhand and thirdhand smoke [9,13,14,15]. (Thirdhand smoke is residual nicotine and other chemicals left on indoor surfaces by tobacco smoke [9].). These studies generally address the experience of secondhand smoke in multiunit, low-income housing. They largely focus on developing interventions and policy to reduce secondhand and thirdhand smoke exposure, and provide little insight into Latinx health beliefs beyond the general agreement that secondhand smoke is harmful. Several note that the Latinx cultural values of *familismo* (responsibility to care for extended family and friends), *respeto* (treating others with respect and not interfering with their personal decisions), *simpatía* (maintaining agreeable social relationships and avoiding direct confrontation), and *personalismo* (relating to others on a personal, friendly level) influenced participants’ willingness to confront smokers or demand smoke-free policies [13,14,15].

For example, Rendón et al. [14] summarize the results of focus groups noting that the participants generally knew the definition and health effects of secondhand smoke, including asthma, other respiratory problems, and an unpleasant quality of life at home. However, they do not provide examples of the definition presented nor delve into how the participants understood secondhand smoke to cause these health effects. The authors note that the major sources of secondhand smoke knowledge were hearsay, healthcare providers, and public service announcements. Baezconde-Garbanati et al. [13] also used focus groups to inform intervention development. Discussions focused on secondhand smoke penetrating apartments, and on how cultural values of *respeto* and *simpatía* made individuals hesitant to ask people not to smoke.

The aim of this qualitative study is to describe beliefs about secondhand smoke and its health affects held by Mexican and Central American immigrants in the United States. It uses an ethnomedicine framework in the analysis of in-depth interviews conducted with Latinx immigrants.

## 2. Materials and Methods

This analysis uses data collected as part of a larger study that designed and evaluated a lay educator science curriculum for limited education adult Latinx immigrants from Mexico and Central America. The content of the science curriculum addressed basic genetic concepts and several environmental health exposures, including secondhand smoke. The Wake Forest School of Medicine Institutional Review Board approved all research procedures (IRB00037972). Participants provided signed informed consent.

### 2.1. Conceptual Framework 

This analysis uses an ethnomedicine approach [16] for understanding health beliefs about secondhand smoke. Ethnomedicine is the comparative study of how those who share culture view disease, how they prevent disease, and how they treat disease. The Explanatory Models of Illness framework developed by Kleinman [17,18] informs this analysis. This framework understands health beliefs to be a combination of conscious and implicit knowledge, and it is particularly well suited for exploring the beliefs and perceptions about common exposures and diseases. In particular, it forces the analyst to focus on how individuals understand the pathophysiology or mechanism of an illness or toxicant. People vary in the content of their explanatory models, but these explanatory models share common features to the extent that persons share a common culture [16]. This framework has been used in the analysis of Latinx immigrant beliefs surrounding other environmental exposures, including pesticides and green tobacco sickness [19,20]; occupational injuries, such as carpal tunnel syndrome [21]; and common diseases, such as diabetes [22].

### 2.2. Participant Recruitment

Participant inclusion criterion were (1) age 18 years and older, (2) born in Mexico or Central America, (3) reside in North Carolina, and (4) speak Spanish. Individuals were excluded if they had a child aged 12 years or younger to prevent tapping into potential participants for a later phase of the parent project. Half of the 60 participants were female and half were male. Only 10% of participants could have completed high school or its equivalent, and at least one-third had to have the equivalent of a sixth grade education or less.

The bilingual study coordinator used a site-based strategy [23] to identify and recruit the potential study participants. Sites, such as community care clinics, stores, community partners, and churches, were used to contact this hard-to-reach population. Because interviewers worked through community partners, the number of potential participants who refused to participate is not known. The study coordinator explained the study in the participant’s preferred language (Spanish or English), including what participation would involve, that participation was voluntary and that they would receive a $25 cash incentive for completing the interview.

### 2.3. Data Collection

The study coordinator completed semistructured, in-depth interviews with the participants from February to August 2017. Interviews took place in the participants’ homes or at another private location of their choice. Interviews were audio recorded and lasted between 40 and 80 min. The interview guide was designed to be appropriate for adults with varying education levels. A section of the interview guide focused on participants’ knowledge and beliefs about secondhand tobacco smoke and its health effects for adults and children. Probes were used throughout the interview to elicit rich, detailed descriptions. Saturation was achieved by the time the 60 interviews were completed, as no new types of responses were emerging.

### 2.4. Data Analysis

A professional service transcribed and translated the recorded interviews from Spanish to English. A bilingual team member checked the transcripts against the audio recordings for accuracy. Team members constructed a dictionary with codes that represented key concepts addressed in the interview guide and emergent themes. They revised definitions for codes as more interviews were conducted. The first few transcripts were assigned to four team members for initial coding to ensure consistency across coders. Codes or tags were attached to relevant sections of the transcripts and discussed by all coders to develop a shared understanding of the codes. One person (who also conducted the interviews) coded all of the transcripts, with the coded transcripts randomly assigned to each of the other three coders for review and summary. Any discrepancies in coding were resolved through discussion by team members. All codes were attached to relevant text within each transcript. Atlas ti v7.0 (Scientific Software Development GMBH, Berlin, Germany) software was used for data management.

The investigators subjected the transcripts/segments to saliency analysis [24,25]. Saliency analysis explores patterns of shared meaning by evaluating recurrent themes based on their frequency of recurrence, participants’ emphasis on the theme, and the explanatory capacity of the theme. Salient themes do not need to be discussed by every participant, but they must be discussed in detail and with emphasis throughout the set of interviews. Salient themes provide insight or explanation of the topic being studied. The salient secondhand tobacco smoke themes that emerged reflected issues surrounding familiarity with secondhand smoke, the relative potency of secondhand smoke, the general health effects of secondhand smoke and the mechanisms for these health effects, and child health effects of secondhand smoke and their mechanisms.

Illustrative quotes are included to support the presentation and interpretation of results. Participant ID and gender (F for female; M for male) are noted for each quote.

## 3. Results

### 3.1. Participant Characteristics 

The participants included 30 women and 30 men. Thirty-nine of the participants were immigrants from Mexico, with 21 being immigrants from the Central American countries of Costa Rica, El Salvador, Guatemala, Honduras, Nicaragua, and Panama. They ranged in age from 19 to 69 years, with 14 (23.3%) aged 18 to 30 years, 19 (31.7%) aged 31 to 40 years, 20 (33.3%) aged 41 to 50 years, and 7 (11.7%) aged 51 to 69 years. Twenty-seven (45.0%) of the participants had six or fewer years of formal education, 28 (46.7%) had from seven through eleven years of formal education, and five (8.3%) had 12 or more years of formal education. Participants were not asked about their smoking status, as this was not a focus of the overall research project. However, from interview responses it appears that most of the participants had never smoked tobacco, a few were former smokers, and a very few were active smokers.

### 3.2. Domains

Participant discussions of secondhand smoke centered on four domains: (1) familiarity and definition of “secondhand smoke”, (2) potency of secondhand smoke, (3) general health effects of secondhand smoke, and (4) child health effects of secondhand smoke. These domains largely reflected questions included in the interview guide that addressed the larger project’s goals. However, participants entirely raised the issue of secondhand smoke potency, as well as specifics aspects of the other three domains.

### 3.3. Familiarity and Definition of “Secondhand Smoke”

The interview guide included specific questions asking participants whether they recognized the terms “secondhand smoke” and “passive smoking.” Most recognized the term secondhand smoke; only one recognized passive smoking. None of the participants were asked about the term “environmental tobacco smoke”, and none volunteered this term.

Most of the participants immediately volunteered a definition in recognizing the term secondhand smoke. These definitions were often simple, for example, “Yes, the smoke when somebody is smoking” (P107 F). Other definitions were more pointed; “Okay, if I smoke and you’re there, if you don’t smoke, it doesn’t matter if you don’t smoke, but you are absorbing it just like a child would be. Your lungs are also filling up with smoke. That’s secondhand cigarettes” (P121 F). The one participant who referred to passive smoking stated, “I don’t smoke and you smoke, and I live with you, so I’m a passive smoker and my children are passive smokers. …In Costa Rica, we call her a passive smoker. You don’t smoke, but you’ve been exposed to it so much that, imagine” (P101 F).

One participant (P158 M) held that most members of the local Latinx community were not familiar or knowledgeable about secondhand smoke, with some confusing it with vehicle exhaust. The interviews indicate that this participant’s perception was incorrect.

Of those participants who did not recognize the terms secondhand smoke or passive smoking, most still provided an accurate definition when they reflected on the terms. One participant stated that she had never heard of secondhand smoke or passive smoking, but then responded to the question, “Do you know what happens if I’m smoking here and you guys are inhaling my smoke?” with the statement “Well, yes, well perhaps you can go outside and smoke, because the kids will get sick. Supposedly, their lungs inhale the cigarette smoke. Maybe you can get cancer or—that’s what I know, but I don’t know if it’s true or not” (P124 F). Another participant stated, “No, I’ve never heard of secondhand smoke. Oh, secondhand smoke, I imagine it’s for smokers, because, I’m thinking, someone else smokes, but I’m still breathing as if I were smoking. I think it’s affecting my lungs in the same way. I think we are the ones inhaling more. I think so, because we’re absorbing and absorbing” (105 F). Finally, a participant noted, “Secondhand smoke. It must be from cigarettes that smell when someone is smoking” (P153 M).

A very few who did not recognize the terms secondhand smoke or passive smoking did not attempt to provide their own definitions. Even these participants made unfavorable comments about secondhand smoke once the interviewer offered a definition.

### 3.4. Potency of Secondhand Smoke

#### 3.4.1. Potency

Although the interviewer never asked participants to judge the relative potency of secondhand smoke, almost two-thirds of the participants (37/60) volunteered that secondhand smoke was worse than primary smoking. Participants’ statements on the potency of secondhand smoke varied.

Yes, I have heard that it is even more harmful than smoking in itself because it is more harmful for the person absorbing it than for the person blowing it. Because it affects our lungs more than the lungs of the smoker. That’s what I have heard. I don’t smoke, right? But they say that—even on TV commercials they say that. That it is more harmful for the person breathing it than for the person smoking the cigarette.

(P107 F)

Secondhand. I don’t know. It’s very, very dangerous. The cigarette is bad but especially the smoke. The one who smokes is bad. But the one breathing it and smelling it is worse. You are breathing in what we don’t want. It’s really bad for me, it gives me a headache. If I’m next to someone who smokes, then I get a bad headache. I don’t smoke, but the smoke is harmful for me. …That’s what I think. It goes to the lungs. I think it’s worse for the one breathing it in. The one who is smoking is enjoying it but the consequences will come later. It’s very dangerous.

(P138 F)

As I understand it, secondhand smoke is the worst. …Because it’s secondhand. [Laughs] I mean, I assume that it’s because it’s something that you aren’t using. …I think that it’s because of—for example, the cigarette. Let’s say I smoke it, so when I consume it, I exhale another chemical, one that’s worse than the one that I’m consuming. So, if you’re not consuming anything, but you’re consuming the waste, let’s call it that, then all that waste is more damaging to you. I think that’s why it’s secondhand smoke.

(P113 M)

Well, I’ve heard them say, for example, if a person is here in a room smoking, the ones that are around him get contaminated. I think it’s worse for them than the smoker. It’s more harmful for them—than the one who exhales, the one who inhales, who inhales the smoke that’s been used, so to speak. And if you breathe it, it’s more toxic than the one who’s smoking.

(P153 M)

Three participants (all women) indicated that smoking is worse than secondhand smoke, or that secondhand smoke is no more dangerous than smoking. For example, one participant stated, “I don’t think it causes as much harm as someone who smokes all the time, because we don’t do it because we want to. We’re not always besides that person. I don’t think there is as much harm as those who do it all the time” (P127 F).

Of the participants who did not recognize the term secondhand smoke, most did not indicate it was a problem greater than primary smoking. However, even in this group, some indicated a belief that secondhand smoke was a greater hazard. One woman stated that she had not heard of secondhand smoke or passive smoking, but still noted in her discussion of the potential health effects of secondhand smoke that, “I don’t know if that’s right. Cancer from the smoke. You don’t even have to smoke it. It’s worse to breathe it in than it is to smoke it. That’s worse” (P135 F).

#### 3.4.2. Mechanism

Participants were unclear about how secondhand smoke was worse than primary smoking. “I don’t know what it contains, but it’s more harmful. …Well, I don’t know what happens [why it is harmful], but—I mean, these are things that I don’t really know too much about, but it’s just the stuff I’ve heard about” (P151 M). However, participants alluded to several mechanisms including smell, inhaled secondhand smoke being more contaminated, and secondhand smoke containing different chemicals.

Several participants referred to “smell” when discussing the potency of secondhand smoke. For example, one participant noted, “…if I were smoking and I blow it out, someone else smells it or inhales it and it can make them sicker than me. [Why?] I don’t know, I’ve just heard that it affects your lungs more quickly because of the nicotine, supposedly” (P146 M). Another noted, “Because we are absorbing it, and it goes to our brain. And you don’t see it. And you are smelling it, and you know that you are getting sick with it” (P114 M).

Other participants indicated that inhaled secondhand smoke was more contaminated than the smoke inhaled by the person who was smoking.

…because the secondhand smoke is already contaminated and you’re inhaling it, and it’s already contaminated by the person who is smoking. So, that’s even worse, because you’re being doubly contaminated, more than smoking it.

(P112 F)

I don’t exactly know the science behind it. I think, simply by expelling the smoke, in a way it’s more contaminated. I don’t know what the reason is. I don’t talk about the subject, I just know secondhand smoke is really bad.

(P131 M)

It’s bad because, you know, the smoke even from a fire is bad, no secondhand smoke it’s because you’re expelling everything to the outside.

(P147 M)

A participant employed as a welder used his work experience in explaining why secondhand smoke was more contaminated.

Well, I work in welding, right? When I’m welding, sometimes I absorb a lot of the smoke. A lot of the smoke. Yes, there’s smoke coming out of it. So, when the smoke comes out, sometimes I—sometimes I get a cold. Sometimes it closes my bronchial tubes. Sometimes I get a cough, sometimes my lungs hurt from all the smoke. So, that’s—let’s say I’m working, if there’s someone else who’s smoking and it turns out that person has something wrong with their lungs, then the smoke that he exhales and if someone else inhales it, it has—it has all the bacteria, all the negative things for the person who is inhaling it. Yes, the person who is exhaling it, it’s not as bad for them as for the person inhaling it. Yes, it’s more toxic for the one inhaling it than the one exhaling it.

(P145 M)

Akin to the idea that exhaled tobacco smoke is more contaminated, a few participants noted that the exhaled smoke was chemically different from the smoke inhaled by the smoker. For example, this participant noted, “It might have different chemicals, I don’t know. But I do know it’s been a long time since they don’t let you smoke inside anymore” (P155 F). Similarly, this participant stated, “Let’s say I smoke it, so when I consume it, I exhale another chemical, one that’s worse than the one that I’m consuming. So, if you’re not consuming anything, but you’re consuming the waste, let’s call it that, then all that waste is more damaging to you” (P113 M).

One participant offered an interesting perspective on the mechanism that makes secondhand smoke more potent.

If you breathe it in. More so, for him, because he inhales it so part of it goes to his lungs and part of it goes to his stomach. But you’re breathing it in, so it goes directly into your lungs. That’s more dangerous. That you breathe it in, but he inhales it so part of it goes to his stomach, not his lungs.

(P108 F)

#### 3.4.3. Sources of Knowledge

Only a few of the participants indicated from whom they learned that secondhand smoke was worse than primary smoking. Most simply noted that they had heard this. Three individuals did note that their source was television. One participant noted that he heard it, “From doctors. Doctors have said so. I’ve heard doctors say that it’s more. …From my cousins who are doctors or other people, friends who say that that smoke is more harmful” (P116 M).

### 3.5. General Health Effects of Secondhand Smoke

#### 3.5.1. Effects

A few participants stated that they did not know the health effects of secondhand smoke. For example, one participant noted, “I honestly don’t [know why it is bad or what happens from breathing the smoke]” (P133 M), and another stated, “Well, I know it’s bad, but I don’t know. I think it causes asthma, it’s one of the causes of asthma in children. That’s it” (P137 M). However, most participants indicated that secondhand smoke had the same health effects as primary smoke: “It’s the same as if he were smoking. It’s the same thing, even though he’s not inhaling it with a cigarette in hand, he’s still inhaling the smoke I’m exhaling, so it’s exactly the same smoke” (P103 F). Participants commonly stated that secondhand smoke affected the lungs in some way, with a few specifying that it caused asthma. Many participants also noted that secondhand smoke caused cancer, including lung cancer. One participant (P134 F) stated that it causes tuberculosis.

#### 3.5.2. Mechanism

Many of the participants made very general statements about how secondhand smoke affected health. For example, one participant discussed how secondhand smoke affected the lungs, potentially through the function of filtering toxins: “I’m sure it does cause you something in your lungs, because that’s where all our air is filtered. It might keep in—they eliminate toxins but they give them to you—[Laughter]—in your body, right?” (P155 F). This participant likened secondhand smoke to smog: “Well, it’s supposedly like smog. That it’s bad, but no—I don’t know how it can affect you. Of course, if you’re locked in a garage with the smog, you will die. …Yes. I think that does the same thing, if you’re inhaling a lot of smog over time, then it can affect your health. Like that” (P106 M).

Some of the participants emphasized smell as the cause of secondhand health effect. “It smells bad, so the smell already affects you. And then the smell causes you to cough and a headache. It is a smell like death; it smells bad in my opinion” (P160 M). This woman was more descriptive, “From what I know, that goes to the lungs and if it goes to the lungs, I imagine it also happens to us. When someone smokes, a lot of smoke expands everywhere and we end up smelling it. You can feel the smoke and we cannot stop breathing, so it obviously goes to the lungs and causes harm” (P127 F).

A few participants suggested that secondhand smoke carries infection or can cause an infection.

Well, first, because of all the chemicals in cigarettes. Those are chemicals that weren’t used before. Secondly, because of illnesses and infections or anything else a person can have and they exhale all that. It’s in the air, they’re particles—and they go everywhere. …Why is it dangerous? It can cause illness, an infection. It can cause asthma. Its smoke that you’re inhaling, you’re absorbing it. It can cause an infection in the lungs, or you can end up—I don’t know, with pneumonia, maybe. Or something like that, you know? …An illness. I don’t know, if it’s in the air, you know, you’re getting it from someone else. And besides getting it from someone else, you’re getting all the ingredients in the cigarette, I would guess.

(P154 M)

Finally, several participants indicated that the health effects of secondhand smoke depends on the characteristics of the individual, particularly their “strength” or “weakness.” As one participant noted,

It depends on your organism. If it’s very weak, then it will be at that moment. If your—what are they called? Those things that—I always tell my kids that their—their strong little soldiers are going to fight against the little soldiers—the good little soldiers are going to fight the bad little soldiers. So, I think it depends on their—what is it called? …The defenses. It depends on how your defenses are. If they are fine, then obviously there is going to be a process for it to develop, but if not, then just in the moment, it’s not going to develop.

(P123 F)

This participant reflected on how life experiences might limit or strengthen the health effects of secondhand smoke.

It depends on the care each one had, the life they lived, too. You know that the way you live is what wears you down, so if you live a good life, you will always have good health, well-cared for, but if you lead a bad life and you’re always drinking and smoking, that quickly wears you down, staying up, all that. That’s not taking care of yourself, so you should take care of yourself and get enough sleep, eat right, good nutrition, and your body will always be all right, but if you’re doing other things that are not good, then that’s not taking care of yourself.

(P147 M)

### 3.6. Child Health Effects of Secondhand Smoke

#### 3.6.1. Child Health Effects

Participants were asked directly whether secondhand smoke was more dangerous for children than for adults. A few of the participants stated that secondhand smoke had the same effect on children and adults. For example, one participant (P136 F) stated, “I think it would be the same because we are breathing in the same thing”, and another (P116 M) said “It affects us all [children and adults] the same.” However, over half of the participants stated that secondhand smoke was more dangerous for children, with a particular emphasis on it causing respiratory health problems, such as asthma.

#### 3.6.2. Mechanism

Participants described a cluster of four linked mechanisms in explaining the reason why secondhand smoke had a greater effect on the health of children over adults. These mechanisms were (1) that children would have more years to experience exposure than would adults, (2) that they were still developing, and (3) therefore, were smaller, weaker, and had a weaker immune system. Finally, (4) the health effects of secondhand smoke depended an individual’s personal strength, with adults, men in particular, being stronger than children.

A few participants noted that children have more years to experience exposure to secondhand smoke and effects. For example, this participant (P103 F) explained, “The more he grows and the more he’s exposed to it, it will affect him more, because if I started smoking at the age of 20, but the difference is that I was 20 and he was two—his body. …he’s just growing, so it’s going to affect him more than me”. Another participant (P107 F) noted, “It always affects us as we grow up. In the long run, later in life, not at that time.”

Many of the participants argued that children are still developing, and are smaller and weaker than adults are, so secondhand smoke will have a greater effect on their health. Participants were very descriptive in discussing the importance of development.

They’re developing. They’re still fragile, super fragile.

(P104 M)

Because they are more tender than we are, because they are younger. We are stronger, more mature—but they are just children. They are more prone to asthma, like I said.

(P111 F)

Because they’re young, so their lungs are still developing.

(P117 F)

It would affect babies, children more. Because they are small and their little lungs aren’t as resistant as ours. They are weaker, they are more fragile.

(P122 F)

The child will be affected more because the child is barely developing and growing. A person like me, it may affect them, but they’ve already developed, but a child who is developing and growing and forming, that would be risky and irreparable, because they would grow up with the harm.

(P131 M)

I think it’s more damaging to a child, because their bodies are very—or what can I say, their liver or organs are very small and weak, so I would imagine that it can be more damaging, right? To the kids than the—well, I mean, it’s also harmful for adults, but I think adults have already been damaged by what they’ve already smoked, but a small child with small organs can be affected more.

(P151 M)

Several participants emphasized that the potential ill effects of secondhand smoke for children result from their weaker immune systems. This is a specific component of their physical development that reflects statements about secondhand smoke causing infection. One participant (P102 F) noted, “It affects the child more. …I think so, because their immune system isn’t as developed as an adult’s”, with another participant (P157 M) stating, “I think that it is more in a child, because they are little, they are more vulnerable. We, as adults, we have a stronger immune system than a baby.” Another participant (P159 M) argued, “I think it is more harmful to children because like I said, because of their development and they don’t have as many antibodies as adults”.

Moreover, similar to discussion about the effects of secondhand smoke on general, adult health, participants indicated that its effects were dependent on the individuals’ strength and their defenses, with adults being stronger than children.

The baby more, I think. …because they’re just beginning their development. An adult is stronger.

(P141 F)

Because each person has their own defenses. So, it can’t be the same. …If she has fewer defenses, then obviously she’ll [daughter] get sick easier. Or the wife.

(P113 M)

The same. Well, you’re older so your body has more defenses. A one-year-old baby doesn’t have many defenses yet. Even if they have a strong body, they don’t have the same ability to tolerate what you tolerate. And it can be worse for the child than for the adult, but in the end it’s the same.

(P145 M)

It affects those who have less defenses, so to speak. Children and the elderly have their defenses, or those who are sick.

(P154 M)

The references to women (“the wife”) and older adults in two of quotes reflects the general belief in stronger, male adults being less susceptible to the harmful effects of secondhand smoke.

## 4. Discussion

Most of these Latinx immigrants were aware of secondhand smoke and provided accurate definitions. They knew it was harmful to their health and believed it was more harmful to the health of children (and older adults). They were not clear as to specific health effects of secondhand smoke in adults, but were clearer about the health effects for children. They were vague about how secondhand smoke affects the health of adults or children and offered some interesting lay explanations. One of the prevailing beliefs was that secondhand smoke was more harmful than actually smoking tobacco products.

At a general level, our results for Latinx immigrants are similar to other studies based on qualitative analysis [9,13,14,15] that report that Latinx participants know what secondhand smoke is and that secondhand smoke is harmful. These other analyses did not delve into participants’ understanding of how secondhand smoke is harmful (its mechanism of action), or how its effects were more harmful for some adults than others, and for children more than for adults.

Participants discussed common mechanisms across adults and children for secondhand smoke resulting in ill health. These mechanisms involved the smell of secondhand smoke, secondhand smoke being similar to an infection and affecting the immune system, and personal strength being protective of secondhand smoke. For children, the issue of personal strength versus personal weakness incorporated the dimensions of children being weaker and more fragile, being smaller, and having weaker immune systems. Children were also seen has having more years than adults to experience secondhand smoke exposure and to develop illness and disease. This latter point is accurate, and additional years of exposure and years for illness to develop are considered in the toxicological and developmental literature when referring to other toxicants [26]. Although not developed by the participants, children’s developing system and faster metabolism also put them at greater health risk from exposure to toxicants, such as secondhand smoke.

The general belief that secondhand smoke exposure is a health hazard, but with lay explanations for how secondhand smoke affects health, is not surprising. This belief is similar to secondhand smoke knowledge among most US residents—native born or immigrants [27]. Delineating these lay understandings and explanations among Latinx immigrants is important for further health education and tobacco intervention development.

It is also important to note that these lay explanations are similar to those described for Latinx immigrant lay explanations of mechanisms for exposure to other environmental toxicants. Quandt et al. [19] used in-depth interviews with Latinx farmworkers to examine beliefs about the health effects of pesticide exposure. They found that farmworkers held that they only needed to worry about pesticides with a strong smell, that the effects of pesticide exposure were discussed in terms of an infection, and that personal strength was important in determining whether pesticide exposure would affect an individual’s health. Similarly, Rao et al. [20] also using in-depth interview data, found that Latinx farmworkers believed, incorrectly, that one factor in susceptibility to green tobacco sickness (acute nicotine poisoning from the transdermal absorption of nicotine that occurs during tobacco cultivation and harvesting) was personal strength. Stronger individuals would not experience green tobacco; those with poor nutrition and who consumed too much alcohol had weak systems and were susceptible to green tobacco sickness.

The belief that secondhand smoke is worse than actually smoking is a novel finding of this study; we have not seen this reported elsewhere. It is highly salient among these Mexican and Central American immigrants: participants volunteered this perspective rather than stating it in response to a leading question in the interview guide; almost two-thirds of the participants volunteered this perspective; and the participants described consistent mechanisms for why secondhand smoke was worse than primary smoking. The mechanisms for this more potent effect of secondhand smoke over primary smoke are consistent with those for health effects, particularly the importance of smell. In addition, the participants stated the belief that the process of producing secondhand smoke made it more contaminated and that it contained different chemicals than does primary smoke.

Other analyses examining Latinx concerns about secondhand and thirdhand smoke for the purpose of developing non-smoking regulations and policy note that the cultural values of *familismo*, *respeto*, *simpatía*, and *personalismo* often limit Latinx individuals’ willingness to confront smokers [13,14,15]. The present analysis did not find evidence of these cultural values, but issues surrounding ways to limit smoking and exposure to secondhand smoke were not discussed in these interviews.

The findings from this analysis should be interpreted in light of this study’s limitations. The study was conducted in a single locale in the southeastern US, so results may not apply to other areas of the country. It recruited a purposive, nonrandom sample. The in-depth interview process results in questions being asked differently from one participant to the next, so results cannot be used to establish specific frequencies or to establish statistical associations. Nonetheless, this study employed a rigorous sampling plan and analysis strategy, recruiting a relatively large number of participants (*n* = 60), drawing upon major dimensions of variability (gender, formal educational attainment, and country of origin) within the Latinx population, and requiring evidence of widespread salience of belief to support all conclusions.

## 5. Conclusions

Mexican and Central American immigrants are generally aware of secondhand smoke and its health effects. Most believe that secondhand smoke poses a greater health risk than primary smoking. They have a consistent lay model of mechanisms by which secondhand smoke affects the health of adults and children, with beliefs that the effects for children are greater. The mechanisms are similar to those reported for other environmental toxicants (pesticides and green tobacco sickness). Understanding these health beliefs informs a framework for further health education and intervention to reduce smoking and secondhand smoke exposure in this vulnerable population 

## References

[1] American Cancer Society (2015). Health Risks of Secondhand Smoke. https://www.cancer.org/cancer/cancer-causes/tobacco-and-cancer/secondhand-smoke.html.

[2] Centers for Disease Control and Prevention (CDC) (2018). Health Effects of Secondhand Smoke. https://www.cdc.gov/tobacco/data_statistics/fact_sheets/secondhand_smoke/health_effects/index.htm.

[3] National Cancer Institute (2018). Secondhand Smoke and Cancer. https://www.cancer.gov/about-cancer/causes-prevention/risk/tobacco/second-hand-smoke-fact-sheet.

[4] Gan W.Q., Mannino D.M., Jemal A. (2015). Socioeconomic disparities in secondhand smoke exposure among US never-smoking adults: The National Health and Nutrition Examination Survey 1988–2010. Tob. Control.

[5] Huang J., King B.A., Babb S.D., Xu X., Hallett C., Hopkins M. (2015). Sociodemographic disparities in local smoke-free law coverage in 10 states. Am. J. Public Health.

[6] Merianos A.L., Jandarov R.A., Choi K., Mahabee-Gittens E.M. (2019). Tobacco smoke exposure disparities persist in U.S. children: NHANES 1999–2014. Prev. Med..

[7] Butz A.M., Halterman J.S., Bellin M., Tsoukleris M., Donithan M., Kub J., Thompson R.E., Land C.L., Walker J., Bollinger M.E. (2011). Factors associated with second-hand smoke exposure in young inner-city children with asthma. J. Asthma.

[8] King B.A., Babb S.D., Tynan M.A., Gerzoff R.B. (2013). National and state estimates of secondhand smoke infiltration among U.S. multiunit housing residents. Nicotine Tob. Res..

[9] Delgado-Rendon A., Cruz T.B., Soto D., Baezconde-Garbanati L., Unger J.B. (2017). Second and thirdhand smoke exposure, attitudes and protective practices: Results from a survey of Hispanic residents in multi-unit housing. J. Immigr. Minor. Health.

[10] Azagba S., Latham K., Shan L. (2020). Sociodemographic differences in secondhand smoke exposure in the United States. Health Educ. Behav..

[11] Windham G.C., Soriano J.W., Dobraca D., Sosnoff C.S., Hiatt R.A., Kushi L.H. (2019). Environmental tobacco smoke exposure in relation to family characteristics, stressors and chemical co-exposures in California girls. Int. J. Environ Res. Public Health.

[12] Green L.W., Kreuter M.W. (2005). Health Promotion Planning: An Educational and Environmental Approach.

[13] Baezconde-Garbanati L.A., Weich-Reushé K., Espinoza L., Portugal C., Barahona R., Garbanati J., Seedat F., Unger J.B. (2011). Secondhand smoke exposure among Hispanics/Latinos living in multiunit housing: Exploring barriers to new policies. Am. J. Health Promot..

[14] Rendón A.D., Unger J.B., Cruz T., Soto D.W., Baezconde-Garbanati L. (2017). Perceptions of secondhand and thirdhand smoke among Hispanic residents of multiunit housing. J. Immigr. Minor. Health.

[15] Savas L.S., Mullen P.D., Hovell M.F., Escoffrey C., Fernandez M.E., Jones J.A., Cavazos J., Gutierrez Monroy J.A.A., Kegler M.C. (2017). A qualitative study among Mexican Americans to understand factors influencing the adoption and enforcement of home smoking bans. Nicotine Tob. Res..

[16] Rubel A.J., Hass M.R., Sargent C.F., Johnson T.M. (1996). Ethnomedicine. Anthropology: Contemporary Theory and Method.

[17] Kleinman A. (1980). Patients and Healers in the Context of Culture: An Exploration of the Borderland between Anthropology, Medicine, and Psychiatry.

[18] Kleinman A. (1988). Illness Narratives: Suffering, Healing and the Human Condition.

[19] Quandt S.A., Arcury T.A., Austin C.K., Saavedra R.M. (1998). Farmworker and farmer perceptions of farmworker agricultural chemical exposure in North Carolina. Hum. Organ.

[20] Rao P., Quandt S.A., Arcury T.A. (2002). Hispanic farmworker interpretations of green tobacco sickness. J. Rural Health.

[21] Arcury T.A., Mora D.C., Quandt S.A. (2015). “…you earn money by suffering pain:” Beliefs about carpal tunnel syndrome among Latino poultry processing workers. J. Immigr. Minor. Health.

[22] Arcury T.A., Skelly A.H., Gesler W.M., Dougherty M.C. (2004). Diabetes meanings among those without diabetes: Explanatory models of immigrant Latinos in rural North Carolina. Soc. Sci. Med..

[23] Arcury T.A., Quandt S.A. (1999). Participant recruitment for qualitative research: A site-based approach to community research in complex societies. Hum. Organ.

[24] Arcury T.A., Quandt S.A., Bell R.A. (2001). Staying healthy: The salience and meaning of health maintenance behaviors among rural older adults in North Carolina. Soc. Sci. Med..

[25] Buetow S. (2010). Thematic analysis and its reconceptualization as ‘saliency analysis’. J. Health Serv. Res. Policy.

[26] Chance G.W., Harmsen E. (1998). Children are different: Environmental contaminants and children’s health. Can. J. Public Health.

[27] Kruger J., Patel R., Kegler M., Babb S.D., King B.A. (2016). Perceptions of harm from secondhand smoke exposure among U.S. adults, 2009–2010. Tob. Induc. Dis..

